# Population Dynamics, Plasma Cytokines and Platelet Centrifugation: Technical and Sociodemographic Aspects of ‘Ovarian Rejuvenation’

**DOI:** 10.3390/clinpract13020039

**Published:** 2023-03-10

**Authors:** E. Scott Sills, Seang Lin Tan

**Affiliations:** 1 Plasma Research Section, FertiGen/CAG, Regenerative Biology Group, San Clemente, CA 92673, USA; 2 Department of Obstetrics & Gynecology, Palomar Medical Center, Escondido, CA 92029, USA; 3 OriginElle Fertility Clinic, Montréal, QC H4A 3J3, Canada; 4 Department of Obstetrics & Gynecology, McGill University Health Centre, Montréal, QC H4A 3J1, Canada

**Keywords:** ovarian stem cells, rejuvenation, infertility, menopause, platelet-rich plasma

## Abstract

While advanced reproductive technologies have attained remarkable increases in sophistication, success, and availability since the 1980s, clinicians always meet a therapeutic impasse when the ovarian reserve reaches exhaustion. Irrespective of fertility aspirations, the decline in and eventual collapse of ovarian estrogen output means that menopause arrives with tremendous physiologic changes and reduced overall productivity. Because more women are gaining in longevity or delaying the age at pregnancy, the number of affected patients has never been larger. As concerns regarding standard hormone replacement therapy and the limitations of IVF are confronted, a workable path to enable primordial germ cell recruitment and de novo oocyte development would be welcome. Proof-of-concept case reports and clinical studies on autologous activated platelet-rich plasma (PRP) or its condensed cytokine derivatives suggest a way to facilitate these goals. However, ovarian PRP faces vexing challenges that place ‘ovarian rejuvenation’ under caution as it enters this therapeutic space. Here, we review key features of experimental human ovarian stem cell isolation/handling and reaffirm the need to harmonize laboratory protocols. Recognizing the regenerative science borrowed from other disciplines, specimen centrifugation, platelet processing, and condensed plasma cytokine enrichment are highlighted here. As the refinement of this rejuvenation approach would promise to reprogram adult ovarian physiology, the disruption of established treatment paradigms for infertility, menopause, and perhaps overall women’s health seems likely. Emerging roles in reproductive biology and clinical practice are thus placed in a broader social and demographic context.

## 1. Introduction

Replacement of terminally differentiated post-natal cells in humans is possible, but in adults this is neither universal nor unlimited. The process was considered sufficiently astonishing in antiquity to merit two mentions in classical literature: Myths of Prometheus and Tityus describe ceaseless liver evulsion followed by overnight renewal [1]. The scientific concept of ‘ovarian rejuvenation’ found modern expression centuries later, also in Greece. With an emphasis on fertility, the Athens IVF group successfully used platelet-rich plasma (PRP) to reset diminished ovarian reserve in poor-prognosis patients [2]. But how might this work? And why would platelets be especially relevant?

It was already known that partial finger regrowth was possible after distal-tip digit amputation during early childhood, although this regenerative capacity is eventually lost over time. Single-cell gene lineage mapping and transcriptomic analyses are providing clarification of this process [3], and platelets represent one place where growth factors and cytokines are highly concentrated. Humans are not the only large animal with platelets where this theme is evident, as seasonal antler regrowth in mature deer provides a more dramatic example of rapid tissue regeneration [4].

Local tissue injury can elicit blastema formation comprising less-differentiated mesenchymal stem cells, where functional (positional) memory persists. Derived from differentiated cells, which received some prior anatomic and/or positional assignment, the blastema gains a capacity to de-differentiate [5,6]. Since stem-like cells have been localized to several post-natal organ settings, it is plausible to anticipate that adult human ovarian tissue might also harbor its own reservoir of such cells [7]. The existence of ovarian cells having stem-like characteristics is now largely settled [8], but separating such oocyte-producing germline cells depends on specified laboratory protocols, how results are interpreted, or which data analysis techniques are used [9,10,11]. Recognizing these points, reproductive science is beginning to detail ovarian PRP preparation and specify treatment techniques. As the information on PRP in the fertility space grows, other medical fields with far greater PRP experience continue to build on their head start to improve clinical response [12,13]. 

## 2. Boundaries and Beginnings

**Figure 1:**
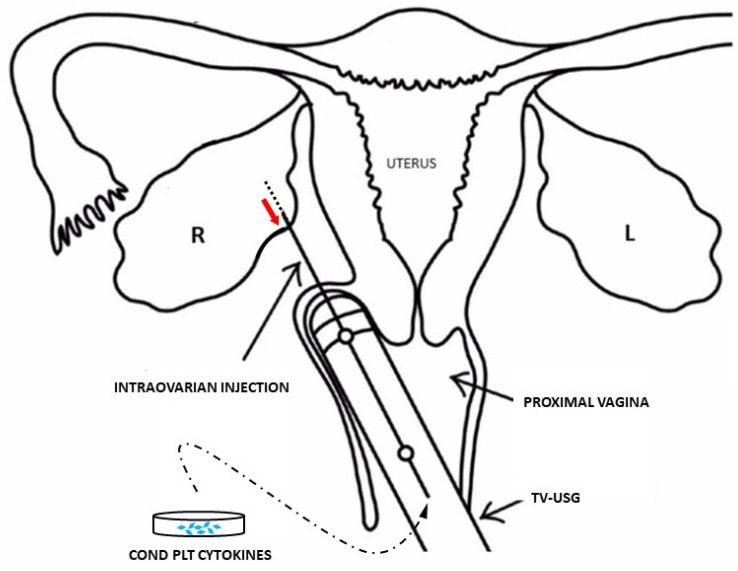
With technical features common to oocyte retrieval/*in vitro* fertilization (IVF), one method is shown for ‘ovarian rejuvenation’ via placement of autologous condensed platelet cytokines (Cond PLT Cytokines) derived from activated platelet-rich plasma. This is placed into the ovarian cortex and subcapsular space by transvaginal ultrasound (TV-USG) upon needle withdrawal (red). Cyclic estradiol and progesterone production is expected to follow, with increased anti-Mullerian hormone output and subsequent emergence of competent *de novo* metaphase II oocytes [14].

Why might cells with stem-like potential reside within the adult human ovary? Latent stem cells situated in terminally differentiated zones have been explained as an evolutionarily conserved adaptation to permit the regeneration of damaged tissues or missing limbs [6]. Working under the theory that such cells may exist in older or nonresponsive adult human ovarian tissue, native cytokines discharged from freshly activated platelets have been surgically inserted into ovarian tissue (see Figure 1). Platelet releasate shares a considerable overlap with so-called ‘Yamanaka factors’, which govern cell differentiation, recruitment, migration, and function [14]. So, how could these cells be confirmed and optimized using this new approach?

For both mice and humans, existence for a beginning (source) point for oocytes found support when small Oct-4+ SSEA+ cells were localized beneath ovarian surface epithelium [15,16]. These discoveries influenced later clinical work, leading to the controlled placement of the full platelet signal array directly to the ovarian subcapsular space via laparoscopy [2]. Cells expressing pluripotency markers termed very small embryonic-like stem cells (VSELs) have since been characterized [17]; within the post-natal ovary, these cells undergo uneven fission to self-regenerate and also yield oogonial stem cells. Of note, they can also exhibit symmetrical division and clonal expansion to produce germ cell nests before meiosis and oocyte differentiation [18,19]. Similar to technical doubts on the separation of ovarian stem cells central to ‘ovarian rejuvenation’, the presence of VSELs was questioned based on specific cytometric gating protocols used for isolation [20].

Against this background, human primordial germ cell and oogonia development was recently achieved from induced pluripotent stem cells where meiosis was induced with subsequent differentiation into primary oocytes via Wnt activation [21]. Bone marrow mesenchymal stem cell research also focusing on Wnt signal transduction has found that hydrostatic pressure change could successfully activate Aggrecan, Col-II, and Sox9 expression when cocultured with platelet-rich fibrin [22]. Although not yet documented in the adult human ovary, Wnt5a with platelet-rich plasma already is known to promote cell differentiation responses elsewhere [23]. Deadbox polypeptide 4 or ‘DDX4′ (mouse vasa homolog/MVH) is an ATP-dependent cytoplasmic RNA helicase absent in somatic tissues but specifically expressed in the germline; it is thus a useful label for oocyte precursors in adult ovaries [24]. Indeed, experience has improved with the use of antibodies specific for CD38, cKIT, EPCAM, ITGA6, PDPN, and TNAP deployed for the separation of primordial germ cells [25,26,27]. 

Beneath the surface epithelium of young adult mouse ovaries, large ovoid cells have been identified akin to germline cells observed in fetal ovaries [28], and immunohistochemical labeling for DDX4 has verified their germline origin [29,30]. Substructural analysis of cellular progenitors by transmission electron microscopy has found large nuclei with euchromatin, thin cytoplasm, and abundant spherical mitochondria [31]. Such work offers insights into how intraovarian PRP might achieve ‘revolutionary’ outcomes for poor-prognosis IVF patients [32] and perhaps even ‘ploidy rescue’ against a history of blastocysts with multiple genetic errors [33].

## 3. Centrifugation for Platelets and Their Stem-Cell Targets

Except for stem-cell sequestration or PRP specimen preparation, centrifugation details are not often critical in IVF benchwork. Standard fertility laboratory processes aim to pellet sperm, debris, or resin, so broad tolerances are allowed. For the processing of PRP samples for intraovarian use, platelets should not be subject to stress >1500 g, and platelet survival is compromised if force exceeds 2200 g. At these speeds, the risk of tube shattering, blood exposure, or other injuries is also increased [34]. In contrast, slow centrifugation is problematic for different reasons. For example, it has been noted [35] that ovarian stem cells might elude detection when suspensions are processed at lower speeds more appropriate to precipitate high-mass components, but not for smaller, less dense targets (e.g., ovarian stem cells). Accordingly, stem cells procured from adult ovarian tissue might be missed [36] when the centrifugation speed is constrained at 300 g [35]. 

Interestingly, ovaries obtained from adult patients undergoing female-to-male gender reassignment surgery (*n* = 16) were used to find ovarian stem cells and a smaller group of comparable age cis-gender patients provided ovarian biopsies collected at cesarian delivery [36]. While gross ovarian follicular density in the main group was similar to histology observed among the C-section patients, all gender reassignment patients had received androgens for up to 7 years before oophorectomy. From microscopic ovary findings detailed in both groups, it was accepted that long-term androgen exposure did not impact ovarian tissue features [36]. However, while the cortical follicle density may look grossly unchanged after prolonged testosterone treatment [37], chronic hyperandrogenic states (*i.e.*, PCOS) may silence histone methyltransferase, triggering dysfunctional gene expression and upregulated mRNA of steroidogenic enzymes including StAR, CYP17A1, and SRD5A1/2 [38,39]. Thus, even if pluripotent stem progenitors in adult ovarian tissue were initially unverified in this study group, this would not necessarily preclude a positive result if receptive cells received different preemptive signaling [14,40]. Mammalian induced pluripotent stem cells have been obtained from somatic cells to generate competent oocytes from embryonic stem cells, and this was extended by later in vitro refinements [40] to establish how somatic cells can be ‘reprogrammed’ to a female germ cell lineage. Given the importance of these issues, precise methods to isolate pluripotent cells as well as the laboratory protocols used for validation deserve close inspection [19,41]. Downstream processes to enrich platelet-derived growth factors or cytokine condensates for intraovarian PRP, especially activation, are likewise critical [42].

## 4. Activation of Fresh Platelets

It has been suggested that fresh platelet activation is an undervalued part of the cytokine release sequence, which is integral for the successful commitment of undifferentiated ovarian stem cells to an oocyte lineage [42]. A recent flow cytometry comparison between platelet products discharged spontaneously vs. after thrombin activation found that thrombin activation can alter platelet releasate composition [43]. While the clinical ovarian tissue response to the PRP product *en toto* or its condensed plasma cytokines is only now being investigated, others have revealed how PRP applied to injured tissues can significantly increase local cAMP levels to decrease inflammation and improve the redox status [44]. The cAMP-mediated process appears to augment PGC-1α expression, which in turn boosts the mitochondrial function [45,46]. This agrees with other work which emphasized relations across reduced tissue metabolism, poor follicular oxygenation, and impaired ovarian function [47]. Despite the role of cAMP documented in platelet operation [48], the biomolecular mechanisms coordinating synthesis and hydrolysis of platelet cAMP in an ovarian context after PRP injection await further study.

It should be mentioned that reproductive gains after ovarian PRP injection have occasionally been attributed to a ‘needle effect’ itself, hypothesized as rather akin to internal acupuncture. However, if this were correct, then the process of oocyte retrieval would be expected to yield an uptick in ovarian reserve following ovary punctures with IVF. Researchers in Vienna [49] were the first to monitor sequential serum AMH levels over multiple oocyte retrievals, observing that repetitive ovarian punctures may diminish—but not boost—the ovarian reserve, especially among IVF patients with PCOS. Of note, when ovarian PRP responses were classified by baseline platelet concentration independent of age, patients (*n* = 182) with higher platelet count were more likely to show increased post-treatment serum AMH than those with lower baseline platelet levels [50].

## 5. Conclusions

Against the blended backgrounds of reproduction and population, the prospect of using intraovarian PRP to defer menopause or repair fertility has entered the public discourse at an interesting time. The historic prediction of Thomas Malthus (1766–1834) suffered from two fundamental errors—neither the technical advancement nor population growth rate behaved according to the forecast. While reduced fertility brings devastating consequences for the individual patient; this also has population-wide effects by downshifting momentum towards sustained contraction and demographic instability [51,52]. In this way, both the census officer and the fertility expert see the same problem through different lenses. Until recently, fertility control was hailed as a useful social policy [53,54], ostensibly to accelerate regional development and personal capital acquisition [55]. A full understanding of national transfer account data required this outlook to be reconsidered, as fertility above the replacement rate is central to government pension solvency and welfare budgets [56]. 

Operating on a different scale, women’s health individually and population status nationally may thus be viewed as sharing a common upstream ramifying term, as both are connected to ovarian fitness and senescence. The issue recently drew comment in the U.K., where reduced birth rates have contributed not only to a rising mean population age but also to the closure of ~4000 nurseries [57,58].

While infertility and symptomatic menopause are both entangled with the ovarian status, the clinical scope of the latter looms far larger (by orders of magnitude) compared to infertility and miscarriage [59]. Indeed, IVF utilization barely registers in the social background, so it cannot realistically be expected to add anything above low single-figure percentages to any country’s national birth statistics. This does not exempt reproductive biologists from our obligation to improve this important intervention. Borrowing guidance from colleagues in engineering, ‘*Inside every complicated problem is many smaller ones waiting to be noticed*’. Further studies on ovarian function should help define how platelet cytokines influence or coordinate this process.

## Data Availability

Not applicable.
